# In vitro comparison of two titanium dental implant surface treatments: 3M™ESPE™ MDIs versus Ankylos®

**DOI:** 10.1186/s40729-017-0083-5

**Published:** 2017-06-27

**Authors:** Jagjit Singh Dhaliwal, Juliana Marulanda, Jingjing Li, Sharifa Alebrahim, Jocelyne Sheila Feine, Monzur Murshed

**Affiliations:** 1 Faculty of Dentistry, McGill University, Montreal, Quebec Canada; 2 PAPRSB, Institute of Health Sciences, Universiti Brunei Darussalam, Jalan Tungku Link, Gadong, BE1410 Brunei Darussalam; 3 Faculty of Medicine, McGill University, Montreal, Quebec Canada; 4 Shriners Hospital for Children, Montreal, Quebec H4A 0A9 Canada

**Keywords:** Cell culture, Osteoblasts, Implant surface

## Abstract

**Background:**

An ideal implant should have a surface that is conducive to osseointegration. In vitro cell culture studies using disks made of same materials and surface as of implants may provide useful information on the events occurring at the implant-tissue interface. In the current study, we tested the hypothesis that there is no difference in the proliferation and differentiation capacities of osteoblastic cells when cultured on titanium disks mimicking the surface of 3M™ESPE™ MDIs or standard (Ankylos®) implants.

**Methods:**

Cells were grown on disks made of the same materials and with same surface texture as those of the original implants. Disks were sterilized and coated with 2% gelatin solution prior to the cell culture experiments. C2C12 pluripotent cells treated with 300 ng/ml bone morphogenetic protein 2 BMP-2 and a stably transfected C2C12 cell line expressing BMP2 were used as models for osteogenic cells. The Hoechst 33258-stained nuclei were counted to assay cell proliferation, while alkaline phosphatase (ALPL) immunostaining was performed to investigate osteogenic differentiation. MC3T3-E1 cells were cultured as model osteoblasts. The cells were differentiated and assayed for proliferation and metabolic activities by Hoechst 33258 staining and Alamar blue reduction assays, respectively. Additionally, cultures were stained by calcein to investigate their mineral deposition properties.

**Results:**

Electron microscopy showed greater degree of roughness on the MDI surfaces. Nuclear counting showed significantly higher number of C2C12 cells on the MDI surface. Although immunostaining detected higher number of ALPL-positive cells, it was not significant when normalized by cell numbers. The number of MC3T3-E1 cells was also higher on the MDI surface, and accordingly, these cultures showed higher Alamar blue reduction. Finally, calcein staining revealed that the MC3T3-E1 cells grown on MDI surfaces deposited more minerals.

**Conclusions:**

Although both implant surfaces are conducive for osteoblastic cell attachment, proliferation, and extracellular matrix mineralization, cell proliferation is higher on MDI surfaces, which may in turn facilitate osseointegration via increased ECM mineralization.

## Background

Prosthetic devices are often used as surrogates for missing skeletal and dental elements. These devices are in close contact with the surrounding tissues, and their functionality and stability are critically dependent on the successful integration within the tissue’s extracellular matrix (ECM). The surface of the implanted device directly interacts with cell and extracellular milieu and influences their biological activities affecting the healing of the implant site after the surgery, tissue regeneration, and the formation of an organic interface with cells and ECM proteins.

Dental implants are commonly used to replace missing teeth, and the long-term success of these implants depends on their proper integration with the mineralized bone, a process commonly known as osseointegration [1].

It has been a long-standing challenge to achieve successful osseointegration of implants in older population with poor bone mass and low bone turnover rates. Therefore, an ideal implant should have a surface which is conducive to osseointegration regardless of the implant site, bone quality, and bone quantity. A large body of literature recommends the use of mini dental implants for stabilization of removable partial and complete dentures in selected situations [2].The 3M™ESPE™ mini dental implant (MDI) system makes use of a self-tapping threaded screw design and needs a minimal surgical intervention. Also, small-size implants have been widely used for orthodontic anchorage [3–5], single tooth replacements [6, 7], fixing the surgical guides for definitive implant placement [8], and as transitional implants for the support of interim removable prosthesis during the healing phase of final fixtures [9, 10]. The MDIs have several advantages over the regular implants used for overdentures such as, simpler surgical protocol and minimally invasive surgery, and they can often be loaded immediately [6]. This helps in reducing postoperative distress to the patient and minimizing resorption of the bone during healing [11]. It has been shown that bone healing around immediately loaded transitional implants is not disturbed and causes no bone loss, which represents a solution for patients who have ridge deficiency and who cannot have surgery for medical reasons [12, 13]. Mini dental implants are also cost-effective, and the price of one MDI is 3.5 times lower than that of a standard size mandibular implant [14].

Despite the advantages of the MDI, evidence on their potential for osseointegration and long-term success is lacking. [15–18]. Newer implant systems entering the market must be studied first in vitro and then in vivo with animal models followed by human studies to demonstrate their osseointegration capability.

Modifications of implant surface properties have been shown to have a positive influence on the successful osseointegration of an implant [19–22]. Surface properties such as roughness, topography, and chemistry are strongly related to the biocompatibility of implants [23]. Thus, modulation of these properties can be useful means to improve implant osseointegration in patients with poor bone quality. The most common treatments used for implant surface modifications are acid etching and sandblasting [24–27]. Implants with moderate surface coarseness demonstrate a better bone response than a smoother or rougher surface [28–30]. When an implant is placed in the bone, a series of cell and matrix events takes place. These mainly include host response to the implant material and behavior of the implant in the host tissue, which culminates in an intimate deposition of a new bone on the implant surface [31].

The immediate event after implantation is adsorption of proteins which may facilitate cell attachment [31]. Various studies show that direct osteoblast-implant interactions are critical for proper osseointegration. Cell culture models using osteoblastic cells are being commonly used to study bone-biomaterial interface [32].

In the current study, we examined the proliferation and differentiation characteristics of differentiated C2C12 cells and MC3T3-E1 preosteoblasts on surfaces mimicking the 3M™ESPE™ MDI (test group) and Ankylos® implants. The Ankylos® implant surface was used for comparison as it is a well-established and widely characterized standard implant.

The surfaces of 3M™ESPE™ MDIs are treated to impart roughness which includes sandblasting with aluminum oxide particles, followed by cleaning and passivation with an oxidizing acid [33]. The Ankylos® implant has the FRIADENT plus surface (Dentsply Implants, Mannheim, Germany). It is formed by sandblasting in a temperature-controlled process and acid etching (hydrochloric, sulfuric, hydrofluoric, and oxalic acid) followed by a proprietary neutralizing technique [34]. Considering that both surfaces were sandblasted and acid-treated, we hypothesize that there is no difference in the proliferation and differentiation capacity of osteoblastic cells when cultured on 3M™ESPE™ MDIs and standard implants.

## Methods

### Implant disks

Titanium disks made up with the same materials and surface characteristics as those with the original implants were obtained from the respective manufacturers. Two types of disks were used; the small disks represented 3M™ESPE™ MDI implants, while the large disks represented Ankylos®, Dentsply Friadent implants. A total of 10 disks of each brand were used for the study.

### Cell culture and in vitro mineralization

Disks were sterilized and coated with 2% gelatin solution to facilitate the attachment of cells. MC3T3-E1 and C2C12 cells were purchased from ATCC (Manassas, VA, USA). Recombinant human bone morphogenetic protein 2 BMP-2 was purchased from GenScript (Piscataway, NJ, USA). MC3T3-E1 and C2C12 cells were cultured in alpha-MEM (Invitrogen, Carlsbad, CA, USA) and DMEM (Invitrogen, Carlsbad, CA, USA), respectively. Culture media were supplemented with 10% FBS (PAA, Etobocoke, Ontario, Canada) and 100 U/ml penicillin–streptomycin (Invitrogen, Carlsbad, CA, USA). Cells were grown at 37 °C under 5% CO2 in a humidified incubator. Mineralization of MC3T3-E1 cultures was induced by addition of ascorbic acid (50 µg/ml) and sodium phosphate (4 mM) to the culture medium for 12 days.

### Calcein staining

Cells were fixed with 4% paraformaldehyde. 0.25% calcein (Sigma-Aldrich, Saint Louis, MO, USA), and 2% NaHCO_3_ solution prepared in 0.15 M NaCl was added to the fixed cells and incubated for 5 min at room temperature. After washing once in PBS, H33258 nuclear staining was performed, washed twice in PBS and images were taken using an inverted fluorescence microscope (EVOS FL, Thermo Fisher Scientific).

### Alamar blue

In order to examine cellular viability/metabolic activity, Alamar blue solution (Resazurin sodium salt, Sigma-Aldrich, Saint Louis, MO, USA) was directly added to the medium to 100 μM final concentration. The reduction of Alamar blue was measured at 560 nm (reference wavelength 610 nm) after 5-h incubation at 37 °C using a microplate reader (Infinite 200, Tecan).

### Generation of BMP2 expressing C2C12 cells

C2C12 cells were electroporated together with 0.4 μg of a BMP-2 expression vector (a kind gift from Dr. Katagiri) and 0.1 μg of pCMV-Tag, which expresses a neomycin-resistance gene. Culture medium was supplemented with 300 μg/ml of G418 (Fisher, Pittsburgh, PA, USA) for 9 days. Clones were picked, amplified, and screened by alkaline phosphatase (ALPL; a downstream target for BMP-2 signaling) staining [35].

### Zymography and Western blotting

Protein samples from the transfected cells were prepared in 1× SDS gel-loading buffer (Laemmli buffer) without adding β-mercaptoethanol and quantified using the Pierce™ Coomassie Plus Assay kit (Thermo Scientific, Rockford, IL, USA). Without heat denaturation, equal amount of protein samples (50 μg) were loaded on a 10% SDS-polyacrylamide gel. After electrophoresis, the gel was incubated in NBT/BCIP (Roche, Mannheim, Germany) staining solution until the bands corresponding to ALPL were clearly visible. Same protein extracts upon heat denaturation in the presence of β-mercaptoethanol were used for Western blotting. The primary antibody used for the analysis was anti-actin (Sigma-Aldrich, Saint Louis, MO, USA). An anti-actin antibody raised in rabbit anti-actin (Sigma-Aldrich, Saint Louis, MO, USA). The secondary antibody was anti-rabbit HRP-IgG (Cell Signaling Technology, Beverly, MA, USA).

### Cell proliferation

Nuclear staining was done by H33258 (Sigma-Aldrich, Saint Louis, MO, USA). After washing in PBS, cells grown on the implants were imaged using an inverted fluorescent microscope (EVOS FL, Thermo Fisher Scientific) and cell nuclei were counted.

### Alkaline phosphatase immunostaining and assay

BMP-2-transfected C2C12 cells were fixed in 4% PFA for 15 min, and then blocked with 5% bovine serum albumin (Fisher, Pittsburgh, PA, USA) in Tris buffered saline-0.025%Triton for 30 min at room temperature, followed by overnight incubation with an anti-mouse alkaline phosphatase antibody (R&D systems, Minneapolis, MN, USA). Detection was done by Dylight 488 rabbit anti-goat secondary antibody (Jackson ImmunoResearch, West Grove, PA, USA) with 1-h incubation at room temperature. Fluorescence imaging was performed using an inverted microscope (EVOS FL, Thermo Fisher Scientific). ALPL assay using p-nitrophenyl phosphate was performed as described before [35].

### Scanning electron microscopy

For scanning electron microscopy (SEM), cleaned and sterilized disks in self-sealed pouches were received as such from the respective manufacturers. The disks were carefully mounted on stubs, sputter-coated, and viewed with Carl Zeiss AG-EVO® 40-series scanning electron microscope.

### Statistical analysis

Statistical significance of the differences between the groups was determined using Student’s *t* test. The statistical power was calculated using the Biomath online software (http://www.biomath.info/power/index.html). We analyzed 10 samples for each group (alpha error 0.05), which resulted in a statistical power of 92%.

### Blinding of the investigators

While performing the experiments, JM (first co-author) was not aware of the sources/manufacturers of the disks, which were identified by their size (small and large) only. At the end of the analyses, each disk’s manufacturer was revealed to her by JSD (first co-author).

## Results

### Ring culture technique

**Fig. 1:**
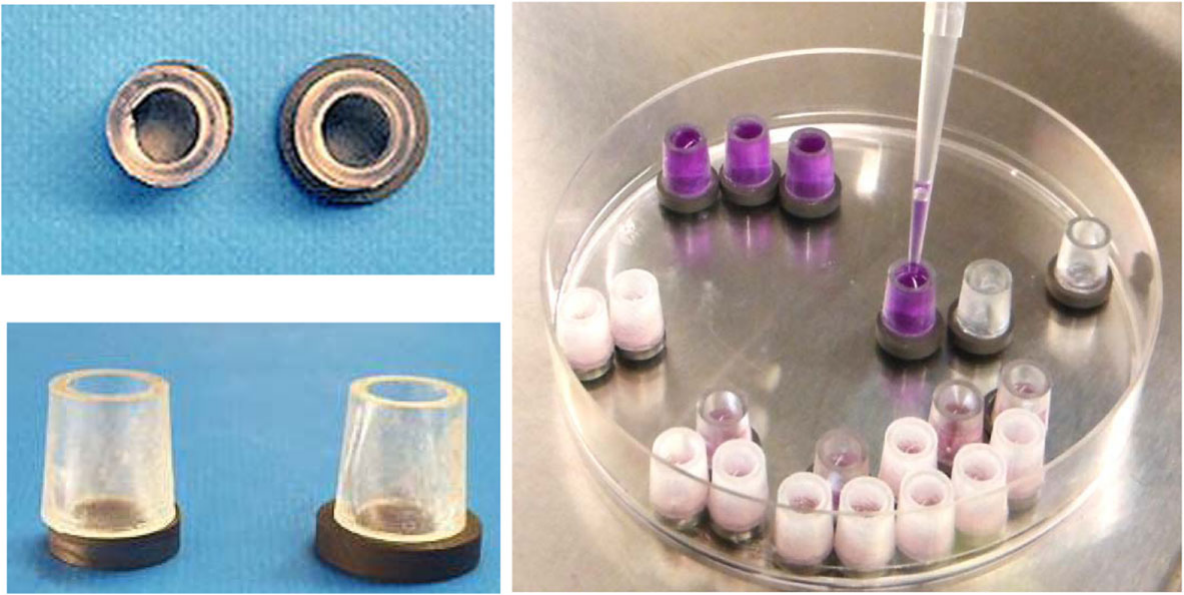
Preparation of specimens. Small disks represent 3M™ESPE™ MDI implants, and large disks represent Ankylos® implants. Note that the attachment of polystyrene rings ensures the area of culture remains constant regardless of the disk size

The variable sizes of the implant disks obtained from two different manufacturers demanded an innovative culture system to ensure equal surface areas on both disks for the cell culture experiments. We achieved this by attaching constant diameter (5 mm) plastic cylinders to the disk surface. Disks were sterilized with absolute alcohol, and polystyrene cloning cylinders (Sigma) were attached onto the disks using vacuum grease. The enclosed surfaces on the disks were then coated with sterile 2% gelatin solution (Fig. 1).

### Increased surface roughness in the 3M™ESPE™ MDIs

**Fig. 2:**
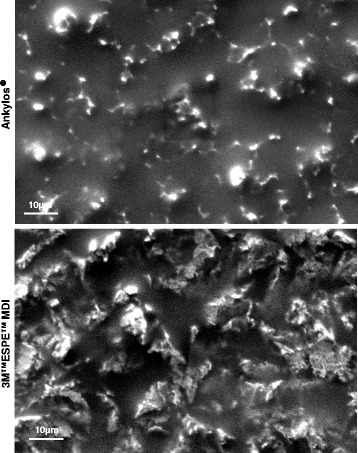
Implant surface characterization under SEM. Increased surface roughness in the 3M™ESPE™ MDI dental implants when compared to Ankylos® implants

Scanning electron microscopy was used in a secondary electron mode under 10-kV acceleration voltage for producing the images to observe the surface topography, and it showed an increased surface roughness in the 3M™ESPE™ MDIs as compared with Ankylos® (Fig. 2).

### Increased proliferation of C2C12 cells grown on 3M™ESPE™ MDI disks

**Fig. 3:**
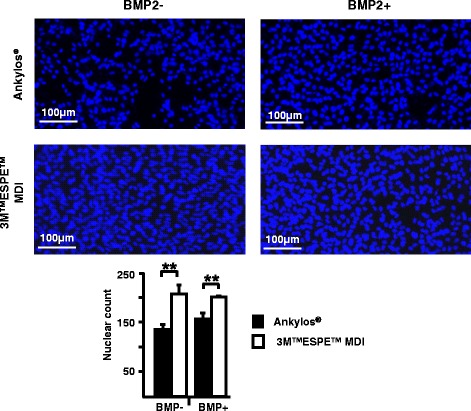
Increased proliferation of C2C12 cells grown on 3M™ESPE™ MDI disks in comparison to the cells grown on the Ankylos® disks untreated and treated with bone morphogenetic protein-BMP-2

We first examined the proliferation of C2C12 cells treated with BMP-2, a pro-osteogenic cytokine, or without BMP-2 treatment on both types of disks. Ten thousand C2C12 cells were plated, and on the following day, the medium was supplemented with 300 ng/ml of BMP-2. Cells were grown for 3 days, stained with the nuclear stain H33258, and imaged using fluorescence microscopy. Counting of cell nuclei revealed an increased proliferation of the C2C12 cells grown on 3M™ESPE™ MDI disks under both conditions, when treated with BMP-2 or without any treatment (Fig. 3).

### Disk type does not affect osteogenic differentiation

**Fig. 4:**
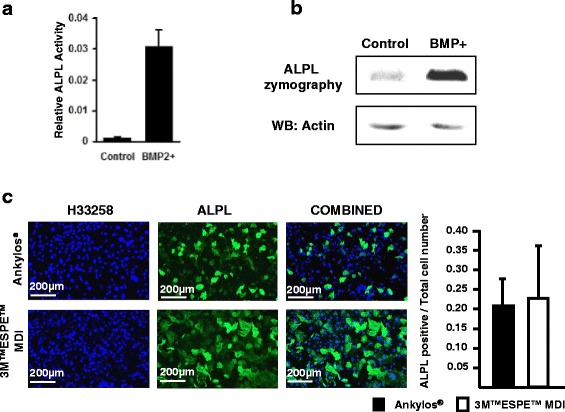
**a** C2C12 cells (control) and pBMP-2-transfected C2C12 cells were seeded on a 24-well plate (50,000 cell/well) and cultured in DMEM medium for 48 h. ALPL assay showing upregulated ALPL activity in the BMP-2-transfected C2C12 cells. **b** Cell extracts of C2C12 cells and pBMP-2-transfected cells were run on a 10% SDS-PAGE under non-denaturing conditions. The gel was then stained with NBT/BCIP solution (upper panel). Western bloting of actin showing the equal protein loading on the gel (*lower panel*). **c** Increased proliferation of C2C12 cells transfected with BMP-2 as well as ALPL activity when seeded on 3M™ESPE™ MDI disks. However, when the number of ALPL-positive cells is normalized to the total cell number, no differences are observed

C2C12 myoblastic cells were transfected with BMP-2. These cells express high levels of ALPL when compared with the control (untransfected) group (Fig. 4a). ALPL zymography showed a more intense band indicating very high expression of functional ALPL protein in the stably transfected cells (Fig. 4b). The transfected cells were then seeded onto each type of disks (15,000 cells/disk) and were cultured for 3 days. Immunostaining using a goat anti-mouse ALPL antibody revealed a significantly higher number of ALPL-positive cells on the 3M™ESPE™ MDI disks in comparison to those on the Ankylos® disks. Interestingly, when the number of ALPL-positive cells was normalized to the total cell number, no differences were observed. This finding suggests that the increase of ALPL-positive cells was not due to an increased cell differentiation, but because of an increased cell proliferation (Fig. 4c).

### Increased proliferation of MC3T3-E1 cells and extracellular matrix mineralization on 3M™ESPE™ MDI disks

**Fig. 5:**
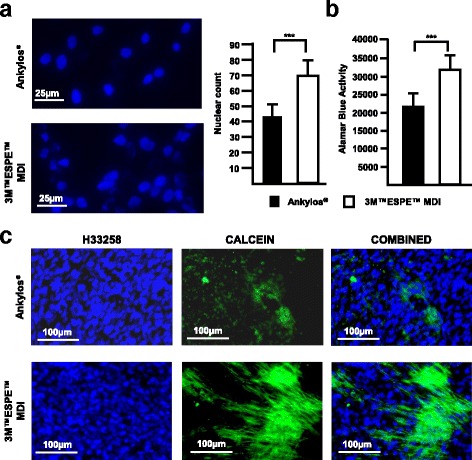
**a** Florescence microscopy showing H33258-stained MC3T3-E1 cells on Ankylos® and 3M™ESPE™ MDI disks. Although equal numbers of cells were plated, after 12 days of culture, more cells were detected on the 3M™ESPE™ MDI disks. **b** Increased Alamar blue® reduction in MC3T3-E1 cells seeded on 3M™ESPE™ MDI disks when compared to cells cultured on Ankylos®. **c** Increased mineral deposition in the MC3T3-E1 cultures on the 3M™ESPE™ MDI disks in comparison to those on the Ankylos® disks as detected by calcein staining

Pre-osteoblastic MC3T3-E1 cells were plated on each implant disk (40,000 cells/disk) and were differentiated with mineralization medium for 12 days. Quantification of cells after nuclear staining by H33258 revealed an increased number of cells on the 3M™ESPE™ MDI disks (Fig. 5a). Measurement of cell viability by the reduction of Alamar blue® after 3 days of culture of MC3T3-E1 cells further supported an increase of cell proliferation on the 3M™ESPE™ MDI disks (Fig. 5b).

In order to assess the ability of the system to promote ECM mineralization, MC3T3-E1 cells were plated at equal densities on each disk type and were grown in the presence of differentiation medium for 12 days. Calcein (binds to calcium salts) staining demonstrated an increased mineral deposition on the surface of the 3M™ESPE™ MDI disks when compared with that on the Ankylos® disks. Increased cell proliferation in the 3M™ESPE™ MDI disks cultures may explain the increase in ECM mineralization (Fig. 5c).

## Discussion

In the current study, we used an in vitro cell culture system to evaluate the biocompatibility of two implant materials with different surface topography. Our objective was to establish the osseointegration potential of MDIs versus an established regular implant. Disks prepared from the implant material were coated with gelatin to grow cells, and proliferation and osteogenic differentiation parameters were evaluated. Considering that the disks obtained from two different sources varied in diameter, we attached 5-mm silicon rings to the surface of both types of disks in order to standardize the culture area. The use of vacuum grease created a leak-proof culture well that enabled us to grow and treat cells for the required period of time. Also, it was possible to use a limited number of disks as the system was easy to clean, disinfect, and reuse.

As cells were grown on metallic surfaces, it was not possible to detect them using light microscopy. This is why we used florescence microscopy to examine the cells and their functional properties once the experiment was complete. Considering that we were unable to routinely examine the live cells on the disks during the culture period, we grew the same number of cells under identical conditions on a plastic cell culture dish enclosed by the same type of culture rings. These cells were evaluated daily using an inverted light microscope, and based on the cell density and the amount of mineral precipitation in this latter culture, we decided to terminate the experiments with the cells grown on the disks.

Two different cell lines were used in our in vitro system: C2C12 and MC3T3-E1 cells. Both of these cell lines were developed from mouse tissues. C2C12 cells are myogenic, but retain the potential to express osteogenic markers under appropriate signaling events. Because of their pluripotency, these cells have been considered as a type of mesenchymal stem cells. It has been shown that when treated with BMPs, these cells readily upregulate many key osteoblast markers including RUNX2, OSX, osteocalcin, and alkaline phosphatase (ALPL) [35]. In the current study, we used C2C12 cells that were treated with BMP-2 or stably transfected with a BMP-2 expression vector.

MC3T3-E1 cells have been extensively used in numerous cell culture experiments as a model for osteoblasts [36]. Under differentiating conditions, e.g., in the presence of ascorbic acid and β-glycerol phosphate, these cells upregulate the osteogenic markers and, more importantly, promote the deposition of calcium phosphate minerals within and around the collagen-rich extracellular matrix (ECM). In comparison to BMP2-treated C2C12 cells, MC3T3-E1 cells are considered to be at a more advanced stage of differentiation towards the osteogenic lineage [35].

Our cell culture system was compatible with both cell types as evident by the outcome of various functional studies, which include cell adherence, synthesis of alkaline phosphatase, and mineralization of the ECM. However, there was a clear difference in the degree of biocompatibility between the two types of implant surfaces; the 3M™ESPE™ MDI showed higher cell numbers and increased deposition of calcium phosphate minerals in comparison to Ankylos®.

The MDI surface was treated with sandblasting and passivation with an oxidizing acid [33] whereas Ankylos® surface was sandblasted and acid etched [34]. The scanning electron microscopic images showed rougher surface in MDIs in comparison to Ankylos®. The blasting process causes a moderate roughness (1–2 micron) to the implants [33].

The surface chemistry and topography of biomaterials seem to play an important role in the success or failure upon placement in a biological environment [37]. It has been established that alterations on the surface topography enhance the bone implant contact and biomechanical interaction of the interface during early implantation periods [37].

MacDonald et al. have shown that wettability, i.e., hydrophilic surfaces support cell interactions and biological fluids better than the hydrophobic surfaces [38]. It has also been shown that roughening the titanium surface improves hydrophilicity [38]. In addition, many authors have stated that rougher surfaces promote differentiation, growth and attachment of bone cells, and higher production of growth factors and augment mineralization [39–43]. However, an in vitro study has demonstrated that osteoblastic cells attach, spread, and proliferate faster on smooth surfaces than on rough surfaces [44].

ALPL is a late osteogenic marker, which is essential for normal bone mineralization. ALPL-deficient osteoblasts fail to mineralize in culture. Considering that there was no significant difference in the relative ALPL activity in cells grown on two surfaces, it is unlikely that the surface property of the disks affected cell differentiation. This observation does not support the findings of Davies that BMPs, alkaline phosphatase and osteocalcin, the important markers of osteogenic differentiation and bone tissue formation, were express at higher levels on rougher surfaces [45]. In addition to surface topography, surface chemistry is also a very strong variable [46, 47]. Therefore, the different surface chemistry of the implant materials used by Davies and our group might have contributed to this discrepancy. Regardless, there is a general agreement that roughening the implant surface greater than the degree seen by machining only leads to a stronger bone formation as shown in a systematic review [48].

Our data suggest that the increased cell number is the primary reason why cultures grown on 3M™ESPE™ MDI deposited more minerals in comparison to those grown on Ankylos®. Taken together, we reject the null hypothesis, since our data demonstrates that MDIs have a superior surface quality that promotes cell proliferation, facilitating osseointegration. However, this needs to be tested in vivo.

## Conclusions

Our results demonstrate that both implant surfaces are conducive for osteoblastic cell attachment, proliferation, and mineralization. However, 3M™ESPE™ MDI surface shows more pronounced effects on cell proliferation, which may in turn facilitate better osseointegration by enhancing ECM mineralization. Our ongoing research will provide further information on how implant surfaces may affect cell behavior at the implant-tissue interface.
